# Aspects of Quality of Life in Interstitial Lung Disease: Pilot Observational Cross-Sectional Study in a Single Center

**DOI:** 10.2196/64409

**Published:** 2026-05-20

**Authors:** Patrycja Rzepka-Wrona, Marzena Trzaska Sobczak, Adam Barczyk, Szymon Skoczyński

**Affiliations:** 1 Department of Lung Diseases and Tuberculosis, Faculty of Medical Sciences in Zabrze, Medical University of Silesia, Koziolka 1, Katowice, Zabrze, 41-803, Poland, 48 32 37 32 235; 2 Department of Pneumonology, School of Medicine in Katowice, Medical University of Silesia, Katowice, Poland

**Keywords:** quality of life, interstitial lung disease, idiopathic pulmonary fibrosis, connective tissue disease, interstitial pneumonia with autoimmune features

## Abstract

**Background:**

Quality of life (QOL) is an important aspect of every chronic disease, including interstitial lung disease (ILD). QOL is perceived as a significant patient-centered outcome.

**Objective:**

This study aims to identify factors correlating with different aspects of QOL in patients with various ILDs.

**Methods:**

We recruited 57 participants hospitalized in a tertiary care clinical center to this pilot observational cross-sectional study. These included 22 patients with idiopathic interstitial pneumonia (IIP), 19 patients with connective tissue disease–associated ILD (CTD-ILD), and 16 patients with interstitial pneumonia with autoimmune features (IPAF). The Saint George’s Respiratory Questionnaire (SGRQ) and World Health Organization Quality of Life Questionnaire (WHOQOL-BREF) were used to assess QOL, and the Hospital Anxiety and Depression Scale - Modified Version (HADS-M) and Patient Health Questionnaire - 9 (PHQ-9) were used to evaluate depression severity. Functional parameters including forced vital capacity (FVC), forced expiratory volume in 1 second (FEV_1_), transfer lung capacity for carbon monoxide (TLCO), and 6-minute walk distance (6MWD) were assessed. Assessment of QOL was a secondary outcome measure in a multicenter prospective study aimed at determining the characteristics of Polish patients with interstitial pneumonia with autoimmune features.

**Results:**

In each study group, positive correlations existed between the WHOQOL-BREF physical domain score and FEV_1_ % predicted value (*P*=.001) and TLCO % predicted value (*P*=.03). Regardless of diagnosis, higher depression, anxiety, and aggression scores (ie, worse mental health) correlated negatively with multiple domains of QOL measured using the WHOQOL-BREF. Predictors of QOL aspects varied in each study group. In the IPAF group, the TLCO % predicted value was a predictor of QOL expressed as the SGRQ total score (*P*=.005). In the CTD-ILD group, short 6MWD (*P*<.001) and high HADS-M aggression score (*P*=.01) correlated with low QOL (expressed as a high SGRQ total score). In the IIP group, 6MWD (*P*=.002) and PHQ-9 scores (*P*<.001) were predictors for SGRQ symptoms score. Gender-based differences were revealed: In all study groups, men had higher scores in the psychological, social, and environmental domains of the WHOQOL-BREF, indicating better QOL, without a statistically significant difference in the physical domain scores between genders. Diagnosis-based differences in the psychological aspects of QOL were also revealed: The QOL psychological domain scores were significantly lower in the CTD-ILD and IPAF groups than in the IIP group, indicating worse QOL (*P*=.01).

**Conclusions:**

QOL is a multifaceted issue with various factors impacting its assessment. 6MWD, TLCO predicted value, and worse functional ability might specifically impact QoL in ILD. Mental health is an important aspect of QOL in the ILD population, as patients with a chronic, potentially life-limiting disease may be more prone to developing depression or anxiety. Assessment of QOL should be taken into account in clinical decision-making and research on chronic diseases, as this patient-related outcome may impact therapeutic decisions and patient compliance.

## Introduction

Interstitial lung disease (ILD) is a broad term encompassing more than 200 clinical entities associated with varying degrees of interstitial inflammation and fibrosis and significant quality of life (QOL) impairment [1-3].

Most available analyses of ILD-related QOL focus on the most prevalent entities (eg, idiopathic pulmonary fibrosis [IPF] or sarcoidosis) [4-6].

Interstitial pneumonia with autoimmune features (IPAF) is a relatively novel entity [7]. To the best of our knowledge, there have been no studies on health-related QOL in this subset of patients with ILD, which is why we wanted to include this patient population in our study.

Patient-reported outcome measures, typically self-applied questionnaires, can be used to assess health-related QOL. Information about patients’ health status is directly obtained from the patients themselves, providing clinicians with patients’ subjective perspectives on disease-associated burden and health status. Other objective measures of ILD severity, such as pulmonary function tests, also provide valuable clinical information on specific aspects of disease severity. However, there are often discrepancies between some objective measures, like results of chest imaging or pulmonary function tests and subjective perceptions on disease burden and how it impacts daily life. Therefore, implementation of patient-related outcome measures may help clinicians realize how patients actually function daily with their disease [8,9].

Development of symptom-specific questionnaires for ILD (eg, cough questionnaires) may be helpful to determine symptom severity and nature on disease burden and advance clinicians’ understanding of self-management strategies and full impact on QOL [10].

Patients with ILD experience a broad range of nonrespiratory symptoms including depression, anxiety, and fatigue that contribute to worse health-related QOL [11].

These symptoms are also associated with adverse outcomes, increased hospital admissions, poor compliance with pharmacotherapy and rehabilitation programs, decreased social life, and premature death [12]. Physical and social aspects of health-related QOL are often affected in patients with ILDs, with key predictors of QOL impairment in this aspect being low physical activity levels and reduced aerobic capacity [13].

The study questions were “Are there functional indicators of QOL common to all types of ILDs?” “Are there gender-based differences between determinants of QOL in patients with ILD?” and “Is perceived QOL dependend on the diagnosis?”

The aim of this observational pilot study was to establish a relationship between functional and psychological symptoms and their impact on QOL. Hopefully, a better understanding of factors correlating with perceived QOL will contribute to improvement in the patient-clinician relationship and compliance with rehabilitation programs and pharmacotherapy, which is of importance while caring for patients who experience side effects of pharmacotherapy and consider discontinuing despite a poor prognosis.

## Methods

### Study Structure

This pilot observational study was conducted in the Department of Pneumonology of Upper Silesian Medical Center, which is a clinical tertiary care hospital. Assessment of QOL was a secondary outcome measure in a multicenter prospective study aimed at determining the characteristics of Polish patients with IPAF [14]. Patient recruitment was conducted in rounds from July 2019 to March 2020 and from April 2021 to December 2021. The pause was caused by the outbreak of the novel coronavirus, when our department was transformed into an infectious diseases ward. We recruited hospitalized adults who had been previously diagnosed with an ILD according to the international guidelines [15-18] .

### Characteristics of Study Participants

We recruited 57 individuals among patients hospitalized in our department: 22 with IIPs (13 patients with IPF and 9 patients with idiopathic nonspecific interstitial pneumonia), 19 with CTD-ILDs, and 16 with IPAF. Patients were hospitalized in order to undergo routine check-ups; therefore, they were not additionally burdened. Patients with deafness or other disorders causing disturbance in verbal communication were excluded. In addition, patients who had previously received treatment for ILD and individuals diagnosed with heart failure whose left ventricle ejection fraction measured during transthoracic echocardiography was ≤45% were excluded. To reduce the impact of mobility issues on 6-minute walk distance (6MWD), patients who used any kind of mobility aid or individuals with cognitive impairment were excluded [19].

### Sample Size Calculation

This article presents the results of a pilot study with a sample size of 57 participants.

According to a simulation by Jenkins and Quintana-Ascensio [20], the minimal sample size can be 8 participants when the groups have low variability. At least one journal now requires a minimum of 5 participants per group for statistical analyses [21]. Ecological studies have been advised to use 10 to 20 participants per predictor [20] or 30 to 45 participants if studying gradients [22].

We aimed to recruit 370 patients for a multicenter study (120 patients in our center) based on an estimated 8000 Polish patients with ILD according to the National Health Fund [23], a confidence level of 95%, and a margin of error of 5%. The number of participants was greater than our initial plans for the pilot study, as many patients are currently hospitalized for inclusion in therapeutic programs for patients with IPF or progressive fibrotic ILD. Moreover, cooperation with a local rheumatological center was also planned.

The minimum sample size was calculated to be 55 participants for linear regression analysis with 1 predictor and assuming a moderate effect (*f*2=0.15), an α of .05, and a test power of 0.8. With 7 predictors with a strong effect (*f*2=0.35), the minimum sample size was calculated to be 49 participants.

### Questionnaires Used in the Study

The degree of dyspnea was measured using the modified Medical Research Council (mMRC) dyspnea scale, with which the severity of dyspnea is assessed on a scale from 0 (dyspneic during strenuous exercise) to 4 (dyspneic when dressing/undressing or unable to leave home due to dyspnea) [24].

Taking into account that no QOL questionnaires are currently validated specifically for ILDs in the Polish population, we used questionnaires designed either for patients with chronic obstructive pulmonary disease (eg, Saint George’s Respiratory Questionnaire [SGRQ]) or individuals undergoing medical interventions (eg, World Health Organization Quality of Life Questionnaire [WHOQOL-BREF]), as these have been validated for use in the Polish-speaking population. CTD-specific questionnaires, such as the Rheumatoid Arthritis Quality of Life Questionnaire, were not used, as they focus specifically on the musculoskeletal symptoms.

### Instruments Used to Assess QOL and Depression and Anxiety Severity

To assess QOL, the SGRQ and WHOQOL-BREF were used. The SGRQ is an instrument with weighted responses that are calculated to produce a score for each component of the questionnaire (ie, symptom severity and frequency, activity impairment due to illness, and impacts including the psychological and social effects of dysfunction) as well as a total score. The SGRQ is scaled from 0 (optimal health) to 100 (worst health) [25]. The WHOQOL-BREF is a 26-item version of the WHOQOL-100 assessment. It is a tool to assess QOL in the physical, psychological, social, and environmental domains. Measures are calculated using an algorithm transforming scores to a 0 to 100-point interval. Higher scores correspond to greater perceived QOL [26].

We chose 2 questionnaires to assess QOL in this population in order to determine whether, similar to QOL assessment in chronic obstructive pulmonary disease, they show comparable reliability.

Data show that the Hospital Anxiety and Depression Scale - Modified Version (HADS-M) and Patient Health Questionnaire - 9 (PHQ-9) vary in their ability to categorize the severity of depression, with the PHQ-9 identifying a greater proportion of patients with moderate or severe depression [27]. Therefore, we decided to include these 2 questionnaires in the study design to determine later, in a full-size study, whether there are differences in depression categorization. As the severity of depression impacts the choice of treatment, the choice of the depression questionnaire may impact therapeutical decisions.

Consent to use these instruments was obtained.

Depression and anxiety were assessed using the HADS-M and PHQ-9.

The HADS-M is a self-administered screening tool for depression, anxiety, and aggression. Scores ranging from 11 to 21 are considered indicative of the presence of a disorder [28].

The PHQ-9 is used to diagnose and determine the severity of depression based on the 9 criteria in the *Diagnostic and Statistical Manual of Mental Disorders* (Fourth Edition; *DSM-IV*), and scores ranging from 20 to 27 are indicative of severe depression [29].

Cough was assessed using a visual analog scale (VAS), which is a linear scale where participants are asked to position the severity of cough on the line from 0 mm to 100 mm (no cough to worst cough, corresponding to subjective intensity of the symptom).

Activity capacity was assessed using the 6-minute walk test performed in a corridor 30 meters long [19]. Individuals using any kind of mobility aids or patients with cognitive impairment were excluded from the study.

### Ethical Considerations

This observational cross-sectional study was performed according to the principles of the Declaration of Helsinki, and the protocol was approved by the Ethics Committee of the Medical University of Silesia (KNW/0022/KB1/130/18/19; date of approval: January 8, 2019). Informed consent forms were provided to every participant, along with information that they could withdraw their consent at any time without any negative impact on their care. Participants gave their consent after after receiving a detailed explanation of the study’s purpose and procedures.

Data collected for this study were anonymous. No compensation was offered to participants in this study.

### Statistical Analysis

Statistical analysis was performed using SPSS version 28.0 (IBM Corp). First, analysis of basic description statistics was performed using the Shapiro-Wilk normality test. To evaluate relationships between the variables, Pearson correlation coefficient and Spearman correlation tests were performed. To compare 2 groups, an independent samples *t* test was performed. To compare 3 groups, univariate analysis was performed. In order to establish which aspects were QOL predictors, multivariate regression analysis was performed. In order to establish the relationships between QOL and pulmonary function test (PFT) parameters, Pearson correlation analysis was performed. In order to determine the relationship between cough VAS and mMRC scores and QOL scores, Spearman correlation analysis was performed. As significance level of .05 was used.

To compare 3 study groups in terms of QOL scores, depression, anxiety, and aggression aspects and activity scores, 1-way ANOVA was performed. To determine the character of differences, a post hoc Bonferroni test was performed.

In order to establish which functional parameters and which aspects of anxiety and depression are QOL predictors, linear regression analysis was performed.

To establish the relationship between treatment duration and QOL and depression and anxiety aspects, Spearman correlation analysis was performed.

## Results

### Group Comparison in Terms of QOL

The analysis revealed significant intergroup differences in QOL psychological domain scores and PHQ-9 and HADS-M depression scores.

The scores for the psychological domain of QOL were significantly lower in the CTD-ILD and IPAF groups than in the IIP group, indicating worse QOL.

However, the post hoc analysis revealed that the score differences in the HADS-M depression domain were irrelevant.

The results are presented in Table 1.

**Table 1.** Intergroup comparison in terms of quality of life (QOL), depression, anxiety, and activity scores (n=57).

Measurement	IPAF^a^ (n=16), mean (SD)		CTD-ILD^b^ (n=19), mean (SD)		IIP^c^ (n=22), mean (SD)		*F* test (*df*)	*P* value	η^2^
WHOQOL-BREF^d^									
Physical domain	43.56 (12.17)		43.21 (15.94)		47.32 (12.45)		0.57 (2,54)	.57	0.02
Psychological domain	39.06 (13.14)		41.32 (16.38)		53.41 (15.96)		5.01 (2,54)	.01^e^	0.16
Social relationships	57.50 (13.71)		56.95 (12.42)		62.86 (14.42)		1.18 (2,54)	.31	0.04
Environment	45.63 (12.74)		49.11 (10.52)		52.55 (9.79)		1.87 (2,54)	.16	0.06
PHQ-9^f^	8.81 (2.90)		9.42 (4.14)		6.73 (2.62)		3.82 (2,54)	.03^g^	0.12
HADS-M^h^									
Depression	8.38 (2.87)		7.84 (3.11)		6.00 (3.01)		3.39 (2,54)	.04^i^	0.11
Anxiety	6.88 (2.96)		7.00 (3.56)		6.27 (3.04)		0.30 (2,54)	.74	0.01
Aggression	1.69 (1.30)		1.79 (1.32)		1.00 (0.93)		2.73 (2,54)	.07	0.09
SQRQ^j^									
Total	51.18 (21.86)		46.25 (20.03)		44.55 (19.18)		0.51 (2,54)	.60	0.02
Symptoms	44.87 (15.79)		38.81 (14.38)		39.02 (15.28)		0.89 (2,54)	.42	0.03
Activity	56.37 (28.19)		50.53 (23.42)		54.98 (24.94)		0.26 (2,54)	.77	0.01
Impacts	51.71 (22.37)		43.03 (19.89)		47.77 (24.67)		0.65 (2,54)	.53	0.02

a IPAF: interstitial pneumonia with autoimmune features.

b CTD-ILD: connective tissue disease–associated interstitial lung disease.

c IIP: idiopathic interstitial pneumonia.

d WHOQOL-BREF: World Health Organization Quality of Life Questionnaire.

e The value in the IIP group was statistically different from the values in both the CTD-ILD and IPAF groups.

f PHQ-9: Patient Health Questionnaire - 9.

g The values in the IIP and IPAF groups were significantly different from the value in the CTD-ILD group, and the value in the IIP group was also significantly different from the value in the IPAF group.

h HADS-M: Hospital Anxiety and Depression Scale - Modified Version.

i Significant differences existed among all 3 groups.

j SGRQ: Saint George’s Respiratory Questionnaire.

#### Models Explaining QOL on the Basis of Depression and Activity Scores

In order to determine which depression aspects (measured by both the HADS-M and PHQ-9) were predictors of QOL, multiple linear regression analysis was performed. Predictor multicollinearity was controlled by a variance inflation factor coefficient <5. All models were well-fitted with data and explained from approximately 30% of the social domain score variance to 85% of the psychological domain score variance. Significant QOL predictors in the physical domain were PHQ-9, SGRQ symptoms, and SGRQ activity scores.

Significant QOL predictors in the psychological domain were depression scores (both HADS-M and PHQ-9) and SGRQ symptoms scores. There was only one significant predictor for QOL in the social domain, namely the SGRQ symptoms score.

The results are presented in Table 2.

**Table 2.** Multivariate linear regression models of quality of life (QOL) aspects for participants in all 3 groups (n=57).

	WHOQOL-BREF^a^ physical domain^b^		WHOQOL-BREF psychological domain^c^		WHOQOL-BREF social relationships^d^		WHOQOL-BREF environment^e^	
	β	*P* value	β	*P* value	β	*P* value	β	*P* value
HADS-M^f^								
Depression	−0.01	.91	−0.53	<.001	−0.25	.18	−0.22	.21
Anxiety	0.14	.23	−0.15	.07	−0.20	.26	0.05	.78
Aggression	−0.02	.79	0.02	.72	−0.20	.18	−0.23	.10
PHQ-9^g^	−0.28	.01	0.27	<.001	0.25	.12	−0.22	.15
SGRQ^h^								
Symptoms	−0.53	<.001	−0.20	.03	−0.57	.006	−0.08	.68
Activity	−0.34	.01	0.08	.40	0.28	.17	−0.23	.22
Impacts	0.13	.16	−0.05	.41	0.15	.30	0.10	.44

a WHOQOL-BREF: World Health Organization Quality of Life Questionnaire.

b *F*_7,49_=20.13, *P*<.001; adjusted *R*2=0.705.

c *F*_7,49_=45.68, *P*<.001; adjusted *R*2=0.848.

d *F*_7,49_=4.26, *P*<.001; adjusted *R*2=0.289.

e *F*_7,49_=5.95, *P*<.001; adjusted *R*2=0.382.

f HADS-M: Hospital Anxiety and Depression Scale - Modified Version.

g PHQ-9: Patient Health Questionnaire - 9.

h SGRQ: Saint George’s Respiratory Questionnaire.

### IPAF Group

A significant predictor of QOL (expressed as the SGRQ total score) was the transfer lung capacity for carbon monoxide (TLCO) % predicted value (*t*_14_=–3.63, *P*=.005). A higher TLCO % predicted value was correlated with lower SGRQ scores.

Higher cough VAS scores were correlated with higher SGRQ symptoms scores. The 2 significant predictors for SGRQ activity scores were cough VAS score (*t*_14_=2.73, *P*=.03) and HADS-M anxiety scores (*t*_14_=–2.36, *P*=.046). Higher cough VAS (*t*_13_=−5.19, *P*<.001) and HADS-M anxiety (*t*_13_=–4.25, *P*=.002) scores were correlated with lower QOL, as expressed by scores in the WHOQOL-BREF physical domain. Higher PHQ-9 scores were correlated with lower scores in the WHOQOL-BREF psychological domain (*t*_14_=–5.49, *P*<.001). The results are presented in Table 3.

**Table 3.** Summary of stepwise regression models explaining domains of quality of life (QOL) in the interstitial pneumonia with autoimmune features (IPAF) group (n=16).

Explanatory variable, step, and predictors			Β^a^	*t* test (*df*)	*P* value	*F* test (*df*)	*P* value	∆*R*^2b^
SGRQ^c^ total score								
Step 1						13.18 (1,14)	.003	0.526
TLCO%^d^			−0.75	−3.63 (14)	.005			
SGRQ symptoms score								
Step 1						10.68 (1,14)	.006	0.468
Cough VAS^e^			0.72	3.27 (14)	.008			
Step 3						14.64 (3,12)	.001	0.788
mMRC^f^			0.31	1.73 (12)	.12			
Cough VAS			0.44	2.73 (12)	.03			
HADS-M^g^ anxiety			0.40	2.36 (12)	.046			
Step 2						34.35 (2.13)	<.001	0.858
Cough VAS			−0.63	−5.19 (13)	<.001			
HADS-M anxiety			−0.51	−4.25 (13)	.002			
WHOQOL-BREF^h^ psychological domain								
Step 1						30.17 (1,14)	<.001	0.726
PHQ-9^i^			−0.87	−5.49 (14)	<.001			
WHOQOL-BREF social relationships								
Step 1						8.65 (1,14)	.01	0.410
mMRC			−0.68	−2.94 (14)	.02			

a B: unstandardized regression coefficient.

b Δ*R*²: delta of the determination coefficient.

c SGRQ: Saint George’s Respiratory Questionnaire.

d TLCO: transfer lung capacity for carbon monoxide.

e VAS: visual analog scale.

f mMRC: modified Medical Research Council.

g HADS-M: Hospital Anxiety and Depression Scale - Modified Version.

h WHOQOL-BREF: World Health Organization Quality of Life Questionnaire.

i PHQ-9: Patient Health Questionnaire - 9.

### CTD-ILD Group

The analysis revealed that higher SGRQ total scores, which indicate worse QOL, were correlated with shorter 6MWD (*t*_16_=–4.20, *P*<.001) and higher HADS-M aggression scores (*t*_16_=2.89, *P*=.01). For QOL according to the SGRQ symptoms score, the predictors were cough VAS (*t*_16_=5.56, *P*<.001) and mMRC (*t*_20_=3.45, *P*=.004) scores; higher symptom scores were correlated with worse QOL, expressed as a higher SGRQ symptoms score. The cough VAS score was also a predictor of the SGRQ activity score (*t*_20_=4.27, *P*<.001). 6MWD and HADS-M aggression score were predictors of SGRQ impacts scores (*P*<.001). The results are presented in Table 4.

**Table 4.** Summary of stepwise regression models explaining domains of quality of life (QOL) in the connective tissue disease–associated interstitial lung disease (CTD-ILD) group (n=19).

Explanatory variable, step, and predictors			Β^a^	*t* test (*df*)	*P* value	*F* test (*df*)	*P* value	∆*R*^2b^
SGRQ^c^ total score								
Step 2						11.59 (2,16)	.003	0.570
6MWD^d^			−0.69	−4.20 (16)	<.001			
HADS-M^e^ aggression			0.48	2.89 (16)	.01			
SGRQ symptoms score								
Step 2						54.07 (2,16)	<.001	0.869
Cough VAS^f^			0.64	5.56 (16)	<.001			
mMRC^g^			0.40	3.45 (16)	.004			
SGRQ activity								
Step 1						18.25 (1,17)	<.001	0.519
Cough VAS			0.74	4.27 (16)	<.001			
Step 2						20.19 (2,16)	<.001	0.706
HADS-M aggression			0.71	5.19 (16)	<.001			
6MWD			−0.59	−4.31 (16)	<.001			
Step 4						28.70 (4,14)	<.001	0.874
Cough VAS			−0.29	−2.30 (14)	.04			
mMRC			−0.61	−4.68 (14)	<.001			
PHQ-9^h^			−0.28	−2.92 (14)	.01			
DLCO^i^			0.27	2.67 (14)	.02			
Step 3						45.34 (3,15)	.002	0.893
HADS-M anxiety			−0.69	−7.19 (15)	<.001			
Cough VAS			−0.36	−4.39 (15)	<.001			
HADS-M aggression			−0.24	−2.38 (15)	.03			
WHOQOL-BREF^j^ social relationships								
Step 1						5.46 (1,17)	<.001	0.218
HADS-M anxiety			−0.52	−2.34 (17)	.03			
Step 2						18.72 (2,16)	<.001	0.689
mMRC			−0.59	−4.04 (16)	.001			
PHQ-9			−0.45	−3.09 (16)	.008			

a B: unstandardized regression coefficient.

b Δ*R*²: delta of determination coefficient.

c SGRQ: Saint George’s Respiratory Questionnaire.

d 6MWD: 6-minute walk distance.

e HADS-M: Hospital Anxiety and Depression Scale - Modified Version.

f VAS: visual analog scale.

g mMRC: modified Medical Research Council.

h PHQ-9: Patient Health Questionnaire - 9.

i DLCO: diffusing capacity of the lung for carbon monoxide.

j WHOQOL-BREF: World Health Organization Quality of Life Questionnaire.

### IIP Group

The mMRC score was a significant predictor of SGRQ total score (*t*_20_=2.44, *P*=.03) and SGRQ activity score (*t*_20_=6.93, *P*<.001).

The 2 significant predictors of SGRQ symptoms scores were 6MWD and PHQ-9 scores (*P*=.003).

The 2 significant predictors of QOL, as expressed using the WHOQOL-BREF physical domain scores, were cough VAS (*P*=.02) and mMRC (*P*=.008) scores. Higher cough VAS (*P*=.001) and HADS-M anxiety (*P*=.04) scores were correlated with lower QOL, as expressed using WHOQOL-BREF psychological domain score.

Table 5 shows the results.

**Table 5.** Summary of stepwise regression models explaining domains of quality of life (QOL) in the idiopathic interstitial pneumonia (IIP) group (n=22).

Explanatory variable, step, and predictors			Β^a^	*t* test (df)	*P* value	*F* test (*df*)	*P* value	∆*R*^2b^
SGRQ^c^ total score								
Step 1						5.93 (1,20)	.002	0.225
mMRC^d^			0.52	2.44 (20)	.03		
Step 3						19.54 (2,19)	<.001	0.766
6MWD^e^			−0.74	−5.10 (19)	<.001			
PHQ-9^f^			0.42	3.29 (19)	.005			
SGRQ activity score								
Step 1						48.02 (1,20)	<.001	0.734
mMRC			0.87	6.93 (20)	<.001			
Step 2						10.22 (2,19)	<.001	0.520
Cough VAS^g^			−0.44	−2.57 (19)	.02			
mMRC			−0.52	−3.03 (19)	.008			
Step 3						13.49 (2,19)	<.001	0.595
HADS-M^h^ anxiety			0.64	−4.07 (19)	.001			
Cough VAS			−0.35	−2.24 (19)	.04			
WHOQOL-BREF^i^ environment								
Step 1						10.7 (1,2)	.002	0.365
PHQ-9			−0.63	−3.28 (20)	.005			

a B: unstandardized regression coefficient.

b Δ*R*²: delta of determination coefficient.

c SGRQ: Saint George’s Respiratory Questionnaire.

d mMRC: Modified Medical Research Council.

e 6MWD: 6-minute walk distance.

f PHQ-9: Patient Health Questionnaire - 9.

g VAS: visual analog scale.

h HADS-M: Hospital Anxiety and Depression Scale - Modified Version.

i WHOQOL-BREF: World Health Organization Quality of Life Questionnaire.

### Determinants of QOL

Regarding the relationships between QOL and PFT parameters in all study groups (n=57), the Pearson correlation analysis revealed a moderate correlation between forced expiratory volume in 1 second (FEV_1_) predicted values and QOL scores in the physical domain of the WHOQOL-BREF questionnaire (*r*=0.30, *P*=.02; *R*=0.25, *P*=.06). There was also a positive weak correlation between TLCO predicted values and QOL scores in the physical domain of the WHOQOL-BREF questionnaire (*r*=0.29, *P*=.03; *R*=0.05, *P*=.70).

There was a weak to strong negative correlation between depression scores and WHOQOL-BREF physical domain scores (*r*=–0.45, *P*<.001). WHOQOL-BREF physical domain scores correlated negatively with all HADS-M domain scores (depression: *r*=–0.45, *P*<.001; anxiety: r=–0.43, *P*<.001; aggression: *r*=–0.28, *P*=.03). WHOQOL-BREF psychological domain scores also correlated negatively with all HADS-M domain scores (depression: *r*=–0.87, *P*<.001; anxiety: r=–0.77, *P*<.001; aggression: *r*=–0.52, *P*<.001). There was a moderate negative correlation between the WHOQOL-BREF social relationships score and HADS-M depression (*r*=–0.47, *P*<.001), anxiety (*r*=–0.43, *P*=.001), and aggression (*r*=–0.35, *P*=.008) scores and SGRQ symptoms score (*r*=–0.42, *P*=.001). There was a negative moderate to strong correlation between the WHOQOL-BREF environment score and HADS-M depression (*r*=–0.57, *P*<.001), anxiety (*r*=–0.49, *P*<.001), and aggression (*r*=–0.48, *P*<.001) scores. The results are presented in Tables6  7.

**Table 6.** Correlation matrix of QOL, depression, and activity (n=57).

Variables	WHOQOL-BREF^a^ physical domain	WHOQOL-BREF psychological domain	WHOQOL-BREF social relationships	WHOQOL-BREF environment	PHQ-9^b^	HADS-M^c^ depression	HADS-M anxiety	HADS-M aggression	SGRQ^d^ total score	SGRQ symptoms score	SGRQ activity score	SGRQ impacts score
WHOQOL-BREF physical domain												
*r*	1	0.57	0.39	0.57	–0.56	–0.45	–0.43	–0.28	–0.61	–0.80	–0.76	–0.36
* P* value	—^e^	<.001	.002	<.001	<.001	<.001	<.001	.03	<.001	<.001	<.001	.005
WHOQOL-BREF psychological domain												
*r*	0.57	1	0.50	0.68	–0.76	–0.87	–0.77	–0.52	–0.50	–0.59	–0.50	–0.30
* P* value	<.001	—	<.001	<.001	<.001	<.001	<.001	<.001	<.001	<.001	<.001	.02
WHOQOL-BREF social relationships												
*r*	0.39	0.50	1	0.45	–0.24	–0.47	–0.44	–0.35	–0.22	–0.42	–0.23	–0.10
* P* value	.002	<.001	—	<.001	.07	<.001	.001	.008	.11	.001	.08	.47
WHOQOL-BREF environment												
*r*	0.57	0.68	0.45	1	−0.57	−0.57	−0.49	−0.49	−0.37	−0.45	−0.45	−0.20
* P* value	<.001	<.001	<.001	—	<.001	<.001	<.001	<.001	.004	.001	<.001	.13
PHQ-9												
*r*	−0.56	−0.76	–0.24	−0.57	1	0.65	0.62	0.51	0.43	0.44	0.43	0.22
* P* value	<.001	<.001	.07	<.001	—	<.001	<.001	<.001	.001	.001	.001	.10
HADS-M depression												
*r*	−0.45	−0.87	−0.47	−0.57	0.65	1	0.73	0.54	0.41	0.45	0.39	0.18
* P* value	<.001	<.001	<.001	<.001	<.001	—	<.001	<.001	.002	<.001	.003	.19
HADS-M anxiety												
*r*	−0.43	−0.77	−0.43	−0.49	0.62	0.73	1	0.49	0.42	0.47	0.46	0.24
* P* value	<.001	<.001	.001	<.001	<.001	<.001	—	<.001	.001	<.001	<.001	.08
HADS-M aggression												
*r*	−0.28	−0.52	−0.35	−0.48	0.51	0.54	0.49	1	0.40	0.26	0.23	0.29
* P* value	.03	<.001	.008	<.001	<.001	<.001	<.001	—	.002	.055	.08	.03
SQRQ total score												
*r*	−0.61	−0.50	−0.22	−0.37	0.43	0.41	0.42	0.40	1	0.78	0.78	0.69
* P* value	<.001	<.001	.11	.004	.001	.002	.001	.002	—	<.001	<.001	<.001
SGRQ symptoms score												
*r*	−0.80	−0.59	−0.42	−0.45	0.44	0.45	0.47	0.26	0.78	1	0.81	0.52
* P* value	<.001	<.001	.001	.001	.001	<.001	<.001	.055	<.001	—	<.001	<.001
SGRQ activity score												
*r*	−0.76	−0.50	−0.24	−0.45	0.43	0.39	0.46	0.23	0.78	0.81	1	0.53
* P* value	<.001	<.001	.08	<.001	.001	.003	<.001	.08	<.001	<.001	—	<.001
SGRQ impacts score												
* r*	−0.36	−0.30	−0.10	−0.20	0.22	0.18	0.24	0.29	0.69	0.52	0.53	1
* P* value	.005	.02	.47	.13	.10	.19	.08	.03	<.001	<.001	<.001	—

a WHOQOL-BREF: World Health Organization Quality of Life Questionnaire.

b PHQ-9: Patient Health Questionnaire - 9.

c HADS-M: Hospital Anxiety and Depression Scale - Modified Version.

d SGRQ: Saint George’s Respiratory Questionnaire.

e Not applicable.

**Table 7.** Spearman correlations between treatment duration and aspects of quality of life (QOL) and depression (n=57).

Variables	Symptom duration	
	*r*_s_	*P* value
WHOQOL-BREF^a^ physical domain	−0.12	.47
WHOQOL-BREF psychological domain	−0.32	.04
WHOQOL-BREF social relationships	−0.42	.008
WHOQOL-BREF environment	−0.24	.13
PHQ-9^b^	0.14	.40
HADS-M^c^ depression	0.34	.04
HADS-M anxiety	0.13	.44
HADS-M agression	0.07	.66

a WHOQOL-BREF: World Health Organization Quality of Life Questionnaire.

b PHQ-9: PHQ-9: Patient Health Questionnaire - 9.

c HADS-M: Hospital Anxiety and Depression Scale - Modified Version.

## Discussion

### Principal Findings

ILDs affect multiple aspects of patients’ lives, impairing QOL proportionately to progression of symptoms [30,31]. Enhancing or maintaining QOL, especially in individuals with noncurable QOL, which affects life expectancy, is an important therapeutic goal [9]. IPAF, as a relative novelty, has been associated with some clinical uncertainty, as data regarding its possible progression are scarce [7,32].

To the best of our knowledge, we are the first to evaluate various aspects of QOL in patients with IPAF compared with patients with other ILDs.

According to Yuan et al [33], individuals with CTD-ILD have a lower QOL as measured by the 36-Item Short Form Health Survey (SF-36) physical component score and SGRQ impact, activity, and total domains in comparison with patients with IIP.

In a study with 131 patients with ILD (CTD-ILD, IPF, sarcoidosis, non-IPF IIP) [34], no significant differences in the prevalences of anxiety and depression between the ILD and IPF groups were found.

Interestingly, our pilot study revealed that psychological aspects of QOL were more significantly impaired in the CTD-ILD and IPAF groups than in the IIP group. We are looking forward to an assessment of patients’ mental health on a larger scale in a full-sized study, and we hope that under-recognition of mental health issues in these populations will be addressed in the future.

Similar to other respiratory diseases, we found gender-related differences in the QOL assessment. In all study groups, men had higher QOL scores than women in the psychological, social, and environmental domains of the WHOQOL-BREF; differences in the physical domain scores were not statistically significant. In a study by Han et al [35] with 221 individuals with IPF, the SF-12 mental health, emotional, and social functioning domain scores were significantly better in men than women.

These findings appear interesting in the light of the fact that men tend to have a worse prognosis and lower transplant-free survival with both IPF and non-IPF ILDs [36]. Moreover, men have an increased risk of faster progression of CTD-ILDs, including rheumatoid arthritis [37] and systemic sclerosis [38].

It remains unclear whether biological, gender-associated, or behavioral aspects influence the course of disease progression and perception of QOL and its various aspects.

As expected, higher depression, anxiety, and aggression scores were associated with worse perceived QOL in the psychological, social, environmental, and physical domains of the WHOQOL-BREF.

In a study with patients with IPF, Lee et al [39] revealed that higher depression and anxiety scores on the HADS questionnaire were associated with higher (worse) SGRQ scores. Many studies with patients with ILD have shown that depression and anxiety are common symptoms in this population and that individuals with ILD have a greater risk of developing both depression and anxiety than the general population [34,39]. Similarly, in our study, 48 patients (48/57, 84%) exhibited borderline depression symptoms on the PHQ-9, and 3 patients (3/57, 5%) scored positively for depression; however, in the HADS-M depression domain, 21 patients (21/57, 37%) had borderline results, and 8 patients (8/57, 14%) had positive results. In the multivariate analysis, HADS-M depression scores and PHQ-9 scores were predictors of the psychological composite of QOL (measured with the WHOQOL-BREF), and the HADS-M depression score was also a predictor of the physical composite of QOL. The high percentage of “borderline depression” results in our study population implies the importance of regular screening for depressive symptoms in the disease course.

Only 3 patients in our study received antidepressants (selective serotonin reuptake inhibitors). Interestingly, there are data suggesting a role of serotonin as a profibrotic agent in myofibrolast differentiation and extracellular matrix deposition in not only the cardiac valves or skin but also interstitial lung tissue [40]. Serotoninergic antagonists are studied as potential antifibrotic agents in progressive fibrosing ILD in the course of rheumatoid arthritis and systemic sclerosis [41].

On the other hand, the low percentage of medicated patients draws our attention to the need for routine mental health assessment in all patients with ILD.

Interestingly, the psychological aspects of QOL in the IPAF group were similar to those in the CTD-ILD group and worse than those in the IIP group.

We revealed positive correlations between the predicted values of FEV_1_ and TLCO and physical health domain scores of the WHOQOL-BREF in all study groups. These findings are corroborated by the results of a study with 229 patients with ILD by Szentes et al [42], who showed that a higher forced vital capacity (FVC) % predicted value was associated with higher QOL scores in all domains. However, to assess QOL, the authors used the King’s Brief Interstitial Lung Disease (K-BILD) questionnaire, which is a different tool developed for this specific population. However, we could not compare the findings in our population, as the K-BILD has not yet been translated into Polish nor validated in the Polish population.

Surprisingly, there were no correlations between the functional parameter predicted values and QOL aspects with the exception of SGRQ total score and the TLCO predicted value.

Our findings are in line with those summarized in a systematic review of QOL studies with patients with IPF by Swigris et al [43], which revealed rather weak correlations between PFT parameters (FVC, TLCO predicted value) and various aspects of QOL. Naturally, health-related QOL perception is a much more intricate and complicated matter than just an outcome of PFT parameters; strong and unambiguous correlations would imply a lack of usefulness of QOL measurement tools: Why assess QOL with a dedicated instrument if PFTs will suffice to describe it?

IPF, as a frequently studied ILD, has a dyspnea prevalence of 90% at the moment of diagnosis [44]. The high dyspnea burden has a great impact on both QOL and mortality [45].

We demonstrated that subjective evaluation of dyspnea (mMRC scores) and anxiety scores (HADS-M) may be important determinants of various aspects of QOL. This is a reminder for health care professionals to evaluate patients’ mental health and work on strategies leading to effective stress management.

Higher scores on the VAS cough scale and mMRC dyspnea scale in our study were associated with worse perceived QOL for all its aspects in all studied groups.

Dyspnea is one of the most common complaints from patients with ILD [46].

In patients with IPF, the Medical Research Council (MRC) dyspnea score is a significant determinant of survival, along with Tiffeneau index and total lung capacity [47].

An understanding of the influence of psychophysiological health determinants on dyspnea sensation in patient with ILD is still limited. Establishing the relationships of associated psychological and physiological factors in dyspnea in hospitalized individuals with ILDs may contribute to effective management of pulmonary rehabilitation and to improved QOL for these patients. Some studies have revealed that both psychological determinants, such as depression or anxiety [48], and physiological determinants, such as cough occurrence and severity, reduced activity capacity and decline in pulmonary function [49] that occur with dyspnea in a population of patients with chronic respiratory diseases. Multiple attempts have been made to establish the associations between these psychophysiological factors and dyspnea among patients with various ILDs (eg, IPF or CTD-ILD), and their findings were contradictory. Ryerson and Berkeley [50] revealed that FVC is an independent determinant of dyspnea, similar to the findings of Londner et al [51]. Interestingly, Ryerson and Berkeley [50] also revealed that depression was an independent dyspnea predictor in 52 participants with ILDs. This was not corroborated by Sanchez et al [52], who reported that anxiety or depression did not contribute to dyspnea sensation in this population. We think that studies with patients with various entities are necessary to clarify the relationships of psychophysiological determinants of dyspnea.

Our study has its limitations. The QOL assessment was a secondary outcome in a study focused on the identification of diagnostic biomarkers in bronchoalveolar lavage fluid in patients with ILD. The results should also be corroborated in larger populations.

### Conclusions

Our study has demonstrated that diagnosis of ILD impacts not only physical but also psychological aspects of QOL. Physical aspects of QOL are far more than a resultant of functional test parameters. Due to the abundance of questionnaires used to assess QOL in chronic diseases, there is a need to validate an ILD-focused questionnaire for the Polish population, which would give an overview about the position of patients with ILD compared with those with other chronic respiratory diseases.

## Abbreviations

6MWD: 6-minute walk distance
CTD-ILD: connective tissue disease–associated interstitial lung disease
*DSM-IV*: *Diagnostic and Statistical Manual of Mental Disorders* (Fourth Edition)
FEV_1_: forced expiratory volume in 1 second
FVC: forced vital capacity
HADS-M: Hospital Anxiety and Depression Scale - Modified Version
IIP: idiopathic interstitial pneumonia
ILD: interstitial lung disease
IPAF: interstitial pneumonia with autoimmune features
IPF: idiopathic pulmonary fibrosis
K-BILD: King’s Brief Interstitial Lung Disease
mMRC: modified Medical Research Council
PFT: pulmonary function test
PHQ-9: Patient Health Questionnaire 9
QOL: quality of life
SF-36: 36-Item Short Form Health Survey
SGRQ: Saint George’s Respiratory Questionnaire
TLCO: transfer lung capacity for carbon monoxide
VAS: visual analog scale
WHOQOL-BREF: World Health Organization Quality of Life Questionnaire
